# Predictive Response Value of Pre- and Postchemoradiotherapy Variables in Rectal Cancer: An Analysis of Histological Data

**DOI:** 10.1155/2016/2164609

**Published:** 2016-01-18

**Authors:** Marisa D. Santos, Cristina Silva, Anabela Rocha, Carlos Nogueira, Eduarda Matos, Carlos Lopes

**Affiliations:** ^1^Department of Surgery, Digestive Surgery Service, Hospital de Santo António, Centro Hospitalar do Porto, Largo Abel Salazar, 4099-003 Porto, Portugal; ^2^Department of Community Health, Instituto de Ciências Biomédicas Abel Salazar, Rua Jorge Viterbo Ferreira, No. 228, 4050-313 Porto, Portugal; ^3^Department of Pathology, Pathological Anatomy Service, Hospital de Santo António, Centro Hospitalar do Porto, Largo Professor Abel Salazar, 4099-003 Porto, Portugal; ^4^Department of Pathology and Molecular Immunology, Instituto de Ciências Biomédicas Abel Salazar, Rua Jorge Viterbo Ferreira, No. 228, 4050-313 Porto, Portugal

## Abstract

*Background*. Neoadjuvant chemoradiotherapy (nCRT) followed by curative surgery in locally advanced rectal cancer (LARC) improves pelvic disease control. Survival improvement is achieved only if pathological response occurs. Mandard tumor regression grade (TRG) proved to be a valid system to measure nCRT response. Potential predictive factors for Mandard response are analyzed. *Materials and Methods*. 167 patients with LARC were treated with nCRT and curative surgery. Tumor biopsies and surgical specimens were reviewed and analyzed regarding mitotic count, necrosis, desmoplastic reaction, and inflammatory infiltration grade. Surgical specimens were classified according to Mandard TRG. The patients were divided as “good responders” (Mandard TRG1-2) and “bad responders” (Mandard TRG3-5). According to results from our previous data, good responders have better prognosis than bad responders. We examined predictive factors for Mandard response and performed statistical analysis. *Results*. In univariate analysis, distance from anal verge and ten other postoperative variables related with nCRT tumor response had predictive value for Mandard response. In multivariable analysis only mitotic count, necrosis, and differentiation grade in surgical specimen had predictive value. *Conclusions*. There is a lack of clinical and pathological preoperative variables able to predict Mandard response. Only postoperative pathological parameters related with nCRT response have predictive value.

## 1. Introduction

Neoadjuvant chemoradiotherapy (CRT) followed by total mesorectum excision (TME) surgery and systemic chemotherapy remains the standard of care for locally advanced rectal cancer (LARC), but only a few patients benefit from this treatment modality. Sauer et al. [1] reported a persistent significant improvement of preoperative CRT versus postoperative CRT on local control, although without overall survival improvement, which is only achieved in situations of good or complete pathological response to CRT [2, 3]. Chemoradiation induces tumor downstaging effect, which increases the probability of a complete resection and a sphincter- preserving surgery, benefiting local control. However, some patients still develop distant and/or local recurrence that compromise survival, particularly those with poor pathological tumor response. The histological response to preoperative CRT has been reported in several studies to be closely related to oncologic outcomes [2, 4–7]. A complete pathologic response (pCR), which is characterized by sterilization of all tumor cells, leads to excellent prognosis and is observed in approximately 10% to 30% of cases [8]. The remaining patients exhibit a spectrum of residual disease, ranging from microscopic tumor cell foci on a background of radiation-induced fibrosis to no response at all [9]. The prognostic relevance of pathological tumor response according to several standard grading criteria has been studied extensively [4, 10–13]. In our previous study, the Mandard system proved to be a good determinant of outcome, when cases were grouped into TRG1+2 (good responders) and TRG3+4+5 (bad responders). This methodology proved superior compared to division in groups of ypCR (TRG1) versus all other (TRG2–5) regarding disease-free survival (DFS) and overall survival (OS) [14]. For this reason it would be of great value to predict nCRT-induced histologic regression and tumor shrinkage. The ability to predict the response, either before treatment or during its early stages, could spare “bad responders” patients the expense and stress of undergoing treatment from which they will derive no benefit. Instead, these patients would be candidates for more intensive treatment strategies. Identifying “bad responders” only in posttreatment may also be an indicator for a different and more intensive adjuvant therapy. Nowadays, individualizing the treatment approach in LARC is a demanding goal to achieve.

The present study aims to analyze predictive factors of Mandard tumor response, trying to identify and characterize the “good” and the “bad responders,” comparing classic histological data obtained from diagnostic biopsies with histological alterations from surgical specimens after treatment.

## 2. Materials and Methods

### 2.1. Patient Population

A single-institution database was queried for consecutive patients with LARC, biopsy-proven rectal adenocarcinoma, who underwent nCRT followed by elective radical surgery with TME with curative intent between January 1, 2003, and December 31, 2013.

*Admission Criteria*. Patients with rectal cancers located at less than 12 cm of distance from the anal verge and clinical stages cT2N+M0 or cT3/4 N0/+M0 are included.

*Exclusion Criteria*. Patients with other diagnosed neoplasia, short course RT, yp stage IV, R1/R2 surgery, and death within 60-day postoperative time are excluded.

All patients receiving nCRT who were operated within 8 weeks after radiotherapy conclusion were included in this analysis. Patients receiving short-course radiation were excluded since no downstaging occurs when immediate surgery is carried out.

Staging included rigid proctoscopy, total colonoscopy, chest, abdominal, and pelvic CT scan, endorectal ultrasound (ERUS), pelvic magnetic resonance image (MRI) (since 2008), and carcinoembryonic antigen (CEA) serum levels.

Diagnostic pretreatment paraffin-embedded biopsies of 152 patients (91%) were available and reviewed by an independent element. All of them were characterized in terms of grade, mitotic index (for 10 fields), necrosis grade (scarce/moderate/marked), inflammatory reaction (scarce/moderate/marked), and desmoplastic infiltration (scarce, moderate, and marked). Tumor biopsy samples classification, including grade, were obtained on worst areas whenever available material contained several areas with neoplasia.

Neoadjuvant CRT protocol included total irradiation of 50.4 Gy in 28 fractions and 5-fluorouracil by infusion pump.

Radical surgery consisted mainly in sphincter saving rectal resection (SSRE) or abdominoperineal resection (APR) both with TME. Regarding operative procedure selection, we considered the distance of the lesion to the anus, the comorbidities of the patient, and the condition of the anal sphincter.

The number of samples taken from the resected specimens was variable, with a mean of 6 paraffin blocks per case. The methodology used was the following:

1. Five samples were from the area with macroscopic lesion (assuming it exists), that is, the same as dealing with a specimen from a patient who has not received neoadjuvant therapy. These included the closest macroscopic approach of the macroscopic lesion to the peritoneal surface and/or the mesorectal excision plane, as appropriate.
2. If no viable tumour was identified within the initial 5 blocks, the whole of the remainder of any macroscopic lesion in additional blocks was included.
3. If no viable tumour was identified within the initial or extra blocks, another three further levels from all of these blocks were taken. If no viable tumour was identified in these sections then complete histological tumour regression was assumed.

All obtained slides were seen and reviewed by the same pathologist. Items observed and registered in the biopsies were subsequently analyzed in the resected specimen and the same criteria adopted.

Standard pathologic tumor staging of the resected specimen was performed in accordance with the guidelines of the American Joint Committee on Cancer (AJCC). Circumferential resection margin (CRM) was scored as positive when cancer cells were within 1 mm of the margin. Evidence of ypCR was defined as an absence of viable adenocarcinoma in the surgical specimen or the presence of lakes of mucus without tumor cells.

The histology of all surgical specimens was reviewed and confirmed by an independent element and were classified based on Mandard tumor regression grading system.

**Figure 1:**
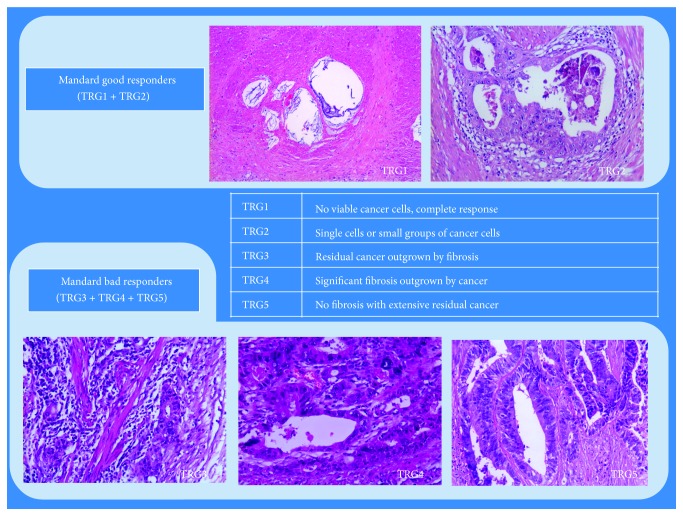
Mandard system.

Patients were divided in two groups according to Mandard TRG system: good responders were defined as Mandard TRG1 or TRG2; bad responders were defined as Mandard TRG3, TRG4, or TRG5 (Figure 1).

Both groups (good responders* versus* bad responders) were used to evaluate outcome results. Operated patients were subjected to adjuvant chemotherapy protocol for 6 months performed preferably with 5-fluorouracil (5-FU) or a combination 5-FU and oxaliplatinum.

Disease recurrence was evaluated according to location: locoregional (LR), systemic (DR), or mixed.

None of the patients were lost for follow-up.

All surviving patients were observed and their current status was confirmed.

Clinical nodal staging may present some inaccuracies; consequently, downstaging evaluation including N stage may introduce some bias, so the term “downstaging” used to describe the efficacy of treatment was defined specifically as T stage downstaging.

Disease-free survival (DFS) was calculated from the date of surgery to the date of progression (local and/or distant), and overall survival (OS) was calculated from the first date of treatment to the date of death or last follow-up.

### 2.2. Statistical Analysis

The survival function was estimated using the Kaplan-Meier method. The difference in survival rates between groups was tested for significance using the log rank test. The significance of differences in proportions was calculated with Chi-square test and the differences in means with Student's* t*-test. A logistic regression analysis was used to assess the independent significance of factors predictive of response, defined as “good” or “bad” responders (TRG1-2/TRG3–5): age; gender; clinical stage; anal–tumor distance; pretreatment CEA; biopsy specimen characteristics: mitotic index, desmoplastic infiltration, inflammatory reaction, and necrosis; CEA post-CRT; surgical procedure; surgical morbidity; pathological stage; circumferential involvement; tumor grade; tumor mitosis number; tumor desmoplastic infiltration; tumor inflammatory reaction; and tumor necrosis in resected specimen were studied.

The statistical analysis was made with SPSS statistical software (version 21.0 for Windows; SPSS Inc., Chicago, IL). All statistical tests were conducted at a two-sided level of significance of 0.05.

## 3. Results

### 3.1. Patient Population

Between January 2003 and December 2013, 186 consecutive patients with LARC were treated with neoadjuvant CRT followed by TME surgery at one single university hospital. We excluded 11 patients with positive radial margin (R1 surgery), 4 patients with yp stage IV, and four deaths within 60 days of postoperative period. In the end, 167 patients were included in the present analysis. Male : female ratio was 1.69 : 1. Median age was 64.6 years (range, 29–83 years). Clinical parameters are summarized in Table 1.

### 3.2. Biopsy Characteristics

The biopsy results are shown in Table 2.

The mitotic index is the number of mitoses for 10 high-powered fields and the cut-off was chosen taking into account the best ratio of sensitivity : specificity. If no 10 high-power fields were present with neoplasia, the number of mitoses was counted in the number of observed fields, reducing the final result to 10 fields.

### 3.3. Surgery

Sphincter saving rectal resection with anastomosis (with or without protective ileostomy) was performed on 107 patients (64.1%). Abdominoperineal resection was performed on 53 patients, and seven patients were subjected to proctectomy with definitive stoma. The perioperative morbidity of the series was of 25,1% with 16 abdominal or pelvic abscesses, 3 anastomotic leaks, 6 reoperations, and 3 readmissions.

### 3.4. Pathology

Stage distribution is shown in Table 3.

The average number of dissected lymph nodes in the surgical specimen was 8.2 (range 0–22).

Circumferential resection margin >1 mm was confirmed in all 167 patients. Response to neoadjuvant therapy is characterized in Table 3.

*Downstaging and Final Pathologic Stage Classification*. T stage downstaging was observed in 67 patients (40.1%). Reduction in T stage by one level was observed in 29 patients (17.4%) and by two or more levels in 38 patients (22.8%). Observations indicating pathologic downstaging are given in Table 3. Ninety-five (56.8%) patients presented pathologic stage one or lower pathologic stage than initial clinical tumor stage. Pathologic complete response (ypCR or Mandard TRG1) was confirmed in 31 patients (18.5%).

*TRG Classification*. The use of Mandard system allowed us to define two groups as previously mentioned: good responders (Mandard TRG1-2) and bad responders (Mandard TRG3–5). Using Mandard system a good response to nCRT was attributed to 85 patients (50.9%) and a bad response was attributed to 82 patients (49.1%).

### 3.5. Clinical Outcome

Table 4 shows long term clinical outcome, relapse of disease, and survival.

With a median follow-up of 59 months (range, 6–139 months), five-year overall survival was 74.6% and pelvic control was 95.8%. Seven patients (4.2%) developed pelvic recurrence (5 isolated and 2 with synchronous metastatic disease) and 22 (12.6%) distant metastases alone. Mandard TRG3–5 (bad responders) is associated with adverse prognosis.

### 3.6. Predictive Factors of Mandard Response

A logistic regression analysis was used to assess the independent significance of pre- and on-treatment variables as predictive factors of Mandard good responders (TRG1-2).  Table 5 shows the 11 variables with predictive value for Mandard response in univariate analysis.

All were related with tumor downstaging and/or with tumor CRT response except the tumor anal distance. The other variables of Tables 1, 2, and 3 have no significant predictive value.

The eleven variables were entered into the Cox-stepwise likelihood ratio. The selection was based upon their statistical significance in univariate analysis and their potential clinical interaction. In the multivariate analysis, after adjustment for the effects of the other variables, tumor with moderated or poor differentiation grade, scarce or moderated necrosis, and >9.5 mitosis number for 10 high-powered fields were more likely to present a bad response (Table 6).

## 4. Discussion

Neoadjuvant CRT followed by curative surgery and adjuvant chemotherapy is used in advanced rectal cancer and the prognosis depends essentially on tumor response. This kind of treatment allows a better pelvic disease control, but better prognosis is achieved only in good responders [2].

The variety of tumor responses has increased the need to find a useful predictive model for the response to CRT in order to identify patients who will really benefit from this multimodal treatment.

In our previous studies Mandard TRG system proved to be fairly accurate, and patients division in good (TRG1-2) and bad responders (TRG3–5) proved effective [10, 14]. These results have been confirmed by other groups [2, 12, 15, 16]. Based on those results it would be important to analyze potential clinical and pathological factors that influence Mandard response.

The influence of clinical parameters in tumor response has been widely studied. In some studies, tumor size, tumor circumferential extent, poor differentiation, mucinous tumor, distance from anal verge, clinical T stage, nodal clinical stage, pretreatment carcinoembryonic antigen (CEA) level, and/or interval of time between surgery and radiotherapy completion were associated with CRT tumor response [17–26]. Magnetic resonance imaging (MRI) and positron emission tomography-computed tomography (PET-CT) also may be useful to predict the response at early stages [27–33]. However, no clinical parameters with prediction value of CRT response have been consistently identified. The results are often different [19, 23, 29, 34, 35]. In our series, distance from anal verge is the only clinical parameter in univariate analysis with predictive value for Mandard response: distal tumors (≤6 cm from anal verge) show a better response according to Mandard. This result is concordant with that described by Das et al. [18]. The opposite is described by Restivo et al. [36]. The delay of surgery after radiotherapy completion from 8 to 12 weeks seems to increase tumor necrosis grade and pathological complete response (ypCR) rate up to 30 to 40% [37–40]. We have 18.5% of ypCR but we cannot study this variable because our patients were operated on average within 8 weeks after radiotherapy conclusion. Pretreatment CEA level is probably the most cited clinical parameter as having tumor response predictive value [18, 20, 21, 41–44]. On our study CEA level, other clinical parameters and the biopsy characteristics analyzed did not predict tumor response to nCRT. Biopsy obtained data, namely, differentiation grade, mitotic index, necrosis, inflammatory, and desmoplastic reaction amount, had no utility to recognize or predict tumor behavior. Tumor hypoxia and proliferative cell activity reduce the effectiveness of both radiation therapy and chemotherapy and are a well-known risk factor for tumor radioresistance. So, it was expected that necrosis, grade, and mitotic number in 10 high-power fields could give any indication of CRT tumor response. However in our series it did not occur with a statistically significant correlation. This may be due to several reasons. The amount of tumor in the biopsy is a very small percentage of the total volume of the whole tumor and, as tumors are heterogeneous, biopsy may not be representative of tumor biology. On the other hand, it suggests that other biologic indicators of response must be investigated, because the ones included in this study are not important for this purpose. Finally it must be remembered that tumor is not the unique actor in the process; local and systemic response of the host, immune, and inflammatory factors also need to be considered.

In our data, all predictive factors found are one way or another related with tumor downstaging and/or nCRT tumor response pathological variables found after treatment. This is an expected result. Pathological TNM stage, tumor downstaging, and CMR of surgical specimen are reflected on nCRT tumor response and are considered in many studies prognostic factors of survival [45–49]. In our study the posttreatment differentiation, feasibility, and proliferative activity of tumor cells show greater impact as predictors of Mandard response (more than pathological TNM stage or tumor downstaging). The presence of accentuated necrosis, mitosis number >9.5 for 10 high-powered fields, and moderate or poorly differentiated grade in resected specimen had a predictive value for Mandard response of 85%. Those variables had a significant predictive value of Mandard response in multivariate analysis.

Thus, the only process for assessing nCRT response is obtained from posttreatment variables. The absence of reliable clinical predictors of response to CRT emphasizes the need to find other factors able to predict response and thus individualize the treatment approach in LARC. On this series the Mandard grade response proved useful to modify the adjuvant treatment plan in patients who were “bad responders” into a more aggressive treatment.

## 5. Conclusions

Mandard system provides an important tool for survival analysis. None of the clinical or the biopsy characteristics assessed had a predictive value of Mandard response, except the distance from anal verge. Only postoperative pathological parameters related with tumor response to chemoradiotherapy have predictive value for Mandard response.

Based on these results it is not yet possible to identify the group of patients who truly benefits from neoadjuvant CRT, but we can identify the group of patients (the Mandard bad responders) that will benefit with a more aggressive adjuvant treatment.

## Figures and Tables

**Table 1:** Clinical parameters.

Variable	*N* (%)
Age	
Mean	64.62
Range	(29–83)
Gender	
Male	105 (62.9)
Female	62 (37.1)
Tumor length	
<4 cm	41 (24.6)
≥4 cm and <6 cm	85 (50.8)
≥6 cm	41 (24.6)
Tumor circumference	
≤1/3	27 (16.2)
>1/3 and ≤1/2	59 (35.3)
>1/2 and ≤2/3	39 (23.4)
>2/3 and ≤3/3	42 (25.1)
Distance from anal verge	
>6 cm	81 (48.5)
≤6 cm	86 (51.5)
CEA pre-CRT	
<5	116 (69.5)
≥5	44 (26.3)
Missing	7 (4.2)
cT stage	
2-3	152 (91.0)
4	15 (9.0)
Clinical stage	
II	76 (45.5)
III	91 (54.5)
CEA post-CRT	
<5	141 (84.4)
≥5	14 (8.4)
Missing	12 (7.2)
Surgery procedure	
AAR/SSO	107 (64.1)
AAP/others	60 (35.9)
Surgery	
Open	129 (77.2)
Laparoscopic	38 (22.8)
Perioperative complications	42 (25.1)
Morbidity	
Abdominal or pelvic abscess	16 (9.5)
Anastomose leak	3 (1.7)
Reoperation	6 (3.5)
Readmission	3 (1.7)

**Table 2:** Biopsies characteristics.

Variable	*N* (%)
*Biopsy*	
Grade	
1	46 (27.5)
2	98 (58.7)
3	8 (4.8)
Missing	15 (9.0)
Mucinous presence	
No	145 (86.8)
Yes	7 (4.2)
Missing/not applied	15 (9.0)
Mitosis number	
≤9.5	34 (20.4)
≥9.6	117 (70.0)
Missing/not applied	16 (9.6)
Inflammatory infiltrate	
Scarce	33 (19.8)
Moderated	66 (39.5)
Marked	52 (31.1)
Missing/not applied	16 (9.6)
Desmoplastic reaction	
Scarce	44 (26.3)
Moderated	80 (47.9)
Marked	27 (16.2)
Missing/not applied	16 (9.6)
Necrosis degree	
Scarce	79 (47.3)
Moderated	40 (23.9)
Marked	32 (19.2)
Missing/not applied	16 (9.6)

**Table 3:** Pathological parameters.

Variable	*N* (%)
ypT stage	
pT0-1	38 (22.8)
pT2–4	129 (77.2)
ypN stage	
pN0	110 (65.9)
pN1-2	57 (34.1)
Pathological stage	
0	58 (34.7)
II	58 (34.7)
III	51 (30.5)
T downstaging	
Yes	67 (40.1)
No	100 (59.9)
Pathological TNM downstaging	
Yes	95 (56.9)
No	72 (43.1)
CRM distance	
>2 mm	159 (95.2)
≤2 and >1 mm	8 (4.8)
Mandard TRG	
TRG1-2 (good responders)	85 (50.9)
TRG3–5 (bad responders)	82 (49.1)
*Other characteristics of resected specimen*	
Grade	
0	32 (19.2)
1	31 (18.6)
2	98 (58.7)
3	6 (3.6)
Mucinous presence	
Yes	41 (24.5)
No	126 (75.4)
Inflammatory infiltrate	
Scarce	60 (35.9)
Moderated	75 (44.9)
Marked	28 (16.8)
Missing	4 (2.4)
Desmoplastic reaction	
Scarce	45 (26.9)
Moderated	64 (38.3)
Marked	51 (30.5)
Missing	7 (4.2)
Necrosis grade	
Scarce	27 (16.2)
Moderated	38 (22.8)
Marked	99 (59.3)
Missing	3 (1.8)
Mitosis number	
≤9.5	73 (43.7)
>9.5	68 (40.7)
Missing	26 (15.6)
Lymphatic permeation	
No	100 (59.9)
Yes	67 (40.1)
Vascular permeation	
No	142 (85)
Yes	25 (15)
Perineural permeation	
No	96 (57.5)
Yes	71 (42.5)
Distal margin	
≥2 cm	108 (65.7)
<2 cm and ≥1 cm	59 (35.3)

**Table 4:** Clinical long term outcome (follow-up median: 59 months; range 6–139).

Variable	
Overall disease recurrence	29 (17.3%)
Local	5 (3%)
Distant	22 (13.2%)
Local and distant	2 (1.1%)
Five-year overall survival	74.6% (se = 3.8%)
Five-year overall survival for “good responders”^*∗*^	88.3% (se = 3.9%)
Five-year overall survival for “bad responders”^*∗*^	58.3% (se = 6.5%)

^*∗*^ *p* < 0.001 (log rank test).

**Table 5:** Predictive value of clinical and pathological characteristics to Mandard response (univariate analysis).

Variables	*n*	Bad responders % (Mandard TRG 3–5)	Odds ratio	*p*
Distance from anal verge				
≤6 cm	86	58.0	1.00	0.017
>6 cm	81	39.5	2.11 (1.14–3.92)	
Mitosis number in resected specimen				
≤9.5	73	32.9	1.00	<0.001
≥9.6	68	77.9	7.21 (3.40–15.32)	
Necrosis grade in resected specimen				
Scarce	27	77.8	10.36 (3.76–28.57)	<0.001
Moderate	38	92.1	34.53 (9.76–122.41)	<0.001
Marked	99	25.3	1.00	<0.001
CRM distance				
≥2 mm	159	45.9	1.00	0.003
<2 and >1 mm	8	100	2.18 (1.84–2.58)	
ypT stage				
0-1	38	7.9	1.00	<0.001
2–4	129	60.5	17.84 (5.21–61.09)	
ypN stage				
0	110	32.7	1.00	
1-2	57	78.9	7.71 (3.64–16.34)	<0.001
Pathological stage				
0-I	58	15.5	1.00	<0.001
II	58	55.2	6.70 (2.78–16.14)	<0.001
III	51	78.4	19.80 (7.47–52.48)	<0.001
T downstaging				
Yes	67	23.9	1.00	<0.001
No	100	65.0	5.92	
Pathological TNM downstaging				
Yes	96	34.4	1.00	<0.001
No	70	67.1	3.90	
Reduction of mitosis number				
Yes	83	41.0	1.00	<0.001
No	52	73.1	3.91 (1.84–8.30)	
Differentiation grade in resected specimen				
0 + 1	52	15.4	1.00	<0.001
2 + 3	115	63.5	9.56	

**Table 6:** Multivariate stepwise model-dependent variable Mandard response: “0” good response and “1” bad response.

Variable	Odds ratio	Confidence interval 95%	*p*
Differentiation grade in resected specimen			
0 + 1	1.00	3.38–32.30	<0.001
2 + 3	10.45		
Necrosis grade in resected specimen			
Marked	1.00	3.04–25.54	<0.001
Scarce/moderate	8.82		
Mitosis number in resected specimen			
≤9.5	1.00	1.52–10.90	0.005
≥9.6	4.07		
